# Auditory experience-dependent cortical circuit shaping for memory formation in bird song learning

**DOI:** 10.1038/ncomms11946

**Published:** 2016-06-21

**Authors:** Shin Yanagihara, Yoko Yazaki-Sugiyama

**Affiliations:** 1 Neuronal Mechanism for Critical Period Unit, Okinawa Institute of Science and Technology (OIST) Graduate University, 1919-1, Tancha, Onna-son, Okinawa 904-0495, Japan

## Abstract

As in human speech acquisition, songbird vocal learning depends on early auditory experience. During development, juvenile songbirds listen to and form auditory memories of adult tutor songs, which they use to shape their own vocalizations in later sensorimotor learning. The higher-level auditory cortex, called the caudomedial nidopallium (NCM), is a potential storage site for tutor song memory, but no direct electrophysiological evidence of tutor song memory has been found. Here, we identify the neuronal substrate for tutor song memory by recording single-neuron activity in the NCM of behaving juvenile zebra finches. After tutor song experience, a small subset of NCM neurons exhibit highly selective auditory responses to the tutor song. Moreover, blockade of GABAergic inhibition, and sleep decrease their selectivity. Taken together, these results suggest that experience-dependent recruitment of GABA-mediated inhibition shapes auditory cortical circuits, leading to sparse representation of tutor song memory in auditory cortical neurons.

## Abstract (web summary)

Juvenile zebra finches learn to sing by memorizing and imitating their tutor's song, yet neural correlates of the tutor song have not been shown. Here the authors show a small subset of higher-level auditory cortex neurons are sharply tuned to the tutor's song and modulated by inhibition and arousal state.

## Graphical abstract

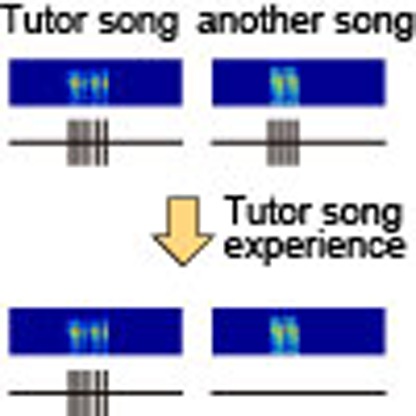

Sensory experience shapes neural circuits in early postnatal development^1^,^2^, contributing to higher cognitive functions, such as learning, in later developmental stages. In rodents, auditory experience-dependent neuronal plasticity has been well-studied^2^,^3^; however, it has not been possible to link that plasticity directly to affective cognitive function. In human speech development, the capacity for phonetic detection depends upon auditory experience in early life^4^, and this experience restricts later language learning. As in human speech acquisition, songbirds learn complex vocalizations based on early auditory experience. In the early sensory learning phase, juvenile zebra finches listen to and memorize a tutor song (normally their fathers' song), and in the subsequent sensorimotor learning phase they match their vocalizations to the memorized tutor song via auditory feedback. This suggests that early auditory experience with the tutor song in the sensory learning phase can shape auditory cortical circuits in the juvenile zebra finch brain, presumably to form a memory of the song, and this memory may regulate later sensorimotor learning in the ‘song system'. However, it remains unclear how and where the tutor song memory is formed in the juvenile brain based on auditory experience.

A growing body of evidence suggests that the caudomedial nidopallium (NCM, a functional homologue of the mammalian higher-level auditory cortex) is one of the possible storage sites of tutor song memories^5^,^6^,^7^. Pharmacological blockade of molecular signalling pathways in higher auditory cortical areas, including the NCM, severely impairs imitation of the tutor song in juvenile birds^8^. In adult birds, lesions in the NCM disrupt tutor song memory-related behaviours, such as recognition of the tutor song^9^ and recovery from vocal changes induced by aversive reinforcement^10^. Furthermore, studies of immediate early gene expression^11^,^12^,^13^ as well as electrophysiological recordings from head-restrained awake birds^14^,^15^,^16^ imply that the NCM may contain neural substrates of the tutor song memory. To date, however, there is no direct electrophysiological evidence to show that neurons in the NCM represent memorized tutor songs in juvenile birds.

In this study we aimed to determine whether and how the memorized tutor song is represented in the songbird auditory cortex, that is, the NCM. We recorded the activity of single neurons in the NCM from behaving juvenile zebra finches before and during song learning. After exposure to a tutor song, a subset of NCM neurons showed highly selective auditory responsiveness to the experienced tutor song. Furthermore, the selectivity of auditory responses was decreased by blocking GABA_A_ receptor-mediated inhibition. These results suggest that auditory experience with the tutor song shapes neuronal circuits in the NCM, presumably through recruitment of GABAergic inhibitory circuits and that the tutor song memory may be represented in a subset of NCM neurons.

## Results

### Tutor experience shapes auditory cortex neuronal selectivity

**Figure 1:**
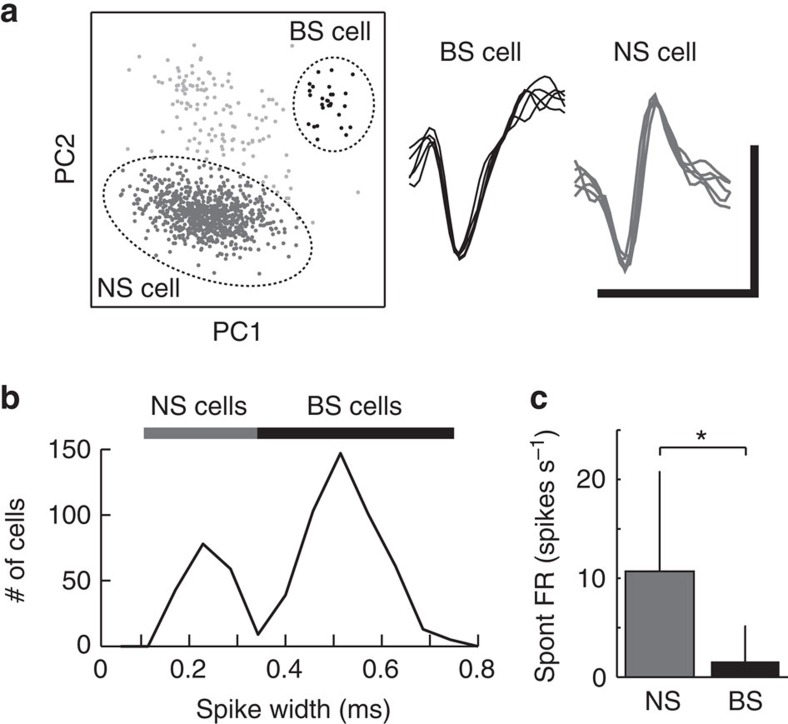
Two distinct neuron populations in the NCM. (**a**) A representative example of spike clusters and spike waveforms of BS (broad spiking) and NS (narrow spiking) neurons. Scale bars, 1 ms (horizontal), 0.2 mV (vertical). (**b**) Spike widths of NCM neurons are bimodally distributed (*n*=658 neurons) BS neuron: 0.50±0.08 ms, *n*=469, NS neuron: 0.21±0.05 ms, *n*=189, (mean±s.d.), Mann–Whitney *U*-test, *P*<0.001. (**c**) Spontaneous firing rate (FR, spikes s^−1^) of NS and BS neurons. Mean±s.d., *n*=189 and 469 for NS and BS neurons, respectively, *P<0.001 by Mann–Whitney *U*-test.

To explore the neuronal representation of tutor song memory, we chronically recorded extracellular single-unit activity from the zebra finch auditory cortical area, that is, the NCM, before and during song learning in the tutored birds (*n*=340 auditory responsive neurons, 50–92 days post hatching, 20 birds), as well as from age-matched isolated control birds (*n*=318 auditory responsive neurons, 55–80 days post hatching, 5 birds) (see Methods section). As shown in a previous study^17^, we found at least two types of neurons in the NCM, broad-spiking (BS) and narrow-spiking (NS) neurons (Fig. 1a), which differed in spike width (Fig. 1b, BS neuron: 0.50±0.08 ms, *n*=469; NS neuron: 0.21±0.05 ms, *n*=189 (mean±s.d.), Mann–Whitney *U*-test, *P*<0.001) as well as in their spontaneous firing rates (Fig. 1c, BS neurons: 1.59±3.54 spikes s^−1^, NS neurons: 10.7±10.1 spikes s^−1^ (mean±s.d.), Mann–Whitney *U*-test, *P*<0.001), as previously reported^17^. Because there were no differences in spike shape between tutored birds and isolated control birds, data from both groups were pooled.

**Figure 2:**
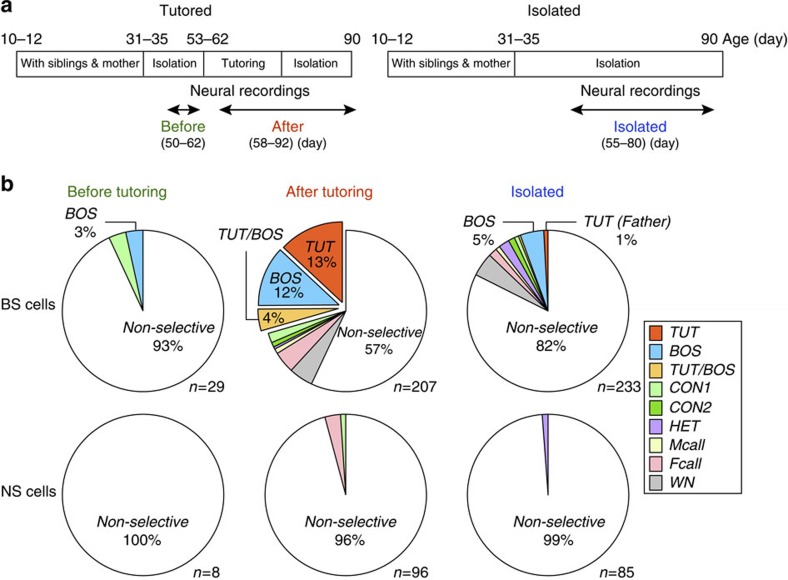
Song-selective neurons emerge after tutoring. (**a**) Timeline of the experimental schedule. (**b**) Percentages of neurons selective for each sound stimulus among BS (top) and NS (bottom) neurons recorded from before tutored (left), after tutored (middle), and isolated birds. Proportion of TUT-selective BS cells in the three groups (before, after and isolated) were significantly different (*χ*^2^-test, *P*<0.001). BOS, bird's own song; CON1, conspecific song-1; CON2, conspecific song-2; Fcall, female call; HET, heterospecific song; Mcall, male call; TUT, tutor song; WN, white noise.

**Figure 3:**
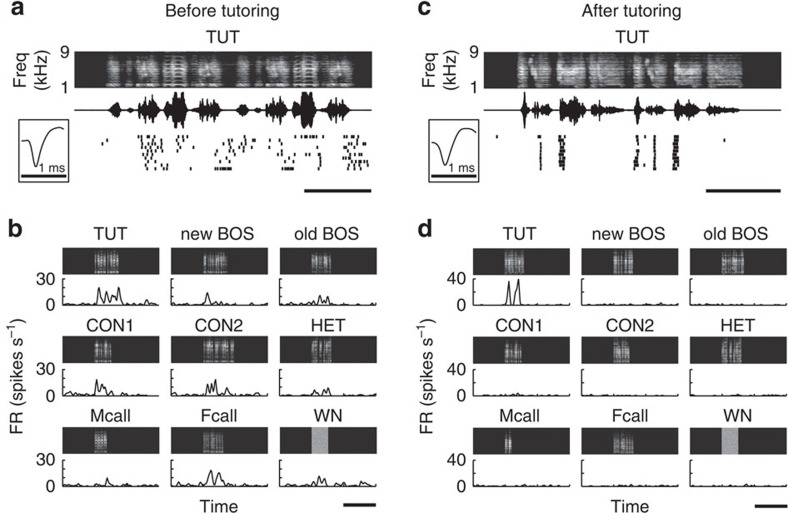
Emergence of tutor song-selective BS neurons after tutoring. A representative non-selective BS neuron recorded before tutoring (59 days of age) (**a**,**b**) and a tutor song (TUT)-selective BS neuron recorded 9 days after tutoring (64 days of age) (**c**,**d**). Neural responses to playback of the tutor song (TUT) are shown as a raster plot (bottom) time-aligned with the TUT spectrogram (top) and oscillogram (middle) (**a**,**c**). Scale bars, 400 ms. Inset: mean spike waveform. Mean firing rate (FR) curves (10 ms, smoothed with a Gaussian kernel) for each auditory stimulus (**b**,**d**). Note that highly reliable and precisely time-locked selective spiking responses to playback of the TUT occur after tutoring. BOS, the bird's own song; CON, conspecific song; Fcall, female call; HET, heterospecific song; Mcall, male call; TUT, tutor song; WN, white noise. Scale bars, 2 s

Interestingly, these neurons showed different auditory response properties, especially after tutor song experience. Various behaviourally relevant sound stimuli (nine stimuli), including a tutor song (TUT), the bird's own song (BOS), conspecific songs (CON), and a heterospecific Bengalese finch song (HET), were presented from a loudspeaker (see Methods section), and neural responses to each stimulus were quantified in terms of response strength (RS), which is the difference between the mean firing rate during the sound stimulus and that during the pre-stimulus baseline period. We further assessed the neuronal discriminability of sound stimuli by calculating *d* values between two sound stimuli^18^. If *d* values for all comparisons between a specific stimulus and other sound stimuli were greater than 0.5, then the neurons were categorized as specific song-selective neurons (see Methods section). Although response habituation with repeated auditory stimuli in the NCM has been reported^14^,^15^,^16^, we did not see it in our recording conditions (chronic recording from freely behaving birds). Neither was it observed for TUT or for other songs in either BS or NS neurons. Before tutoring, both BS (93%) and NS (100%) neurons responded equally well to most sound stimuli (Figs 2b, 3a,b and Supplementary Fig. 2a,b). With 2 weeks of tutor experience, birds could learn from TUT as their song similarity to TUT reached appropriate levels by adulthood^8^,^19^, which is in contrast to the birds that were isolated until adulthood and whose song similarity to TUT remained low (Supplementary Fig. 1a–c). In birds which had tutor song experience, approximately half of BS neurons (43%) displayed highly selective auditory responses to specific sound stimuli (Figs 2b, 3c,d). The emergence of auditory selective BS neurons did not seem to be an exclusive effect of TUT auditory experience, as a significant number of BS neurons in the age-matched control birds that had no TUT experience showed selective responses to specific songs, although the proportions of selective neurons, especially for TUT, were smaller (Fig. 2b, isolated). By contrast, most NS neurons (96%) recorded from tutored birds still responded non-selectively to multiple sound stimuli (Fig. 2b; Supplementary Fig. 2c,d). To further evaluate the response selectivity of each neuron, we quantified the sound stimuli to which each neuron responded using the following equation: selectivity index (SI)=1−(*n*/total number of song stimuli), where *n* is the number of auditory stimuli that drive statistically significant auditory responses (*P*<0.05). As a population, the SI of BS neurons was significantly higher than that of NS neurons, regardless of tutoring experience (Supplementary Fig. 3).

**Figure 4:**
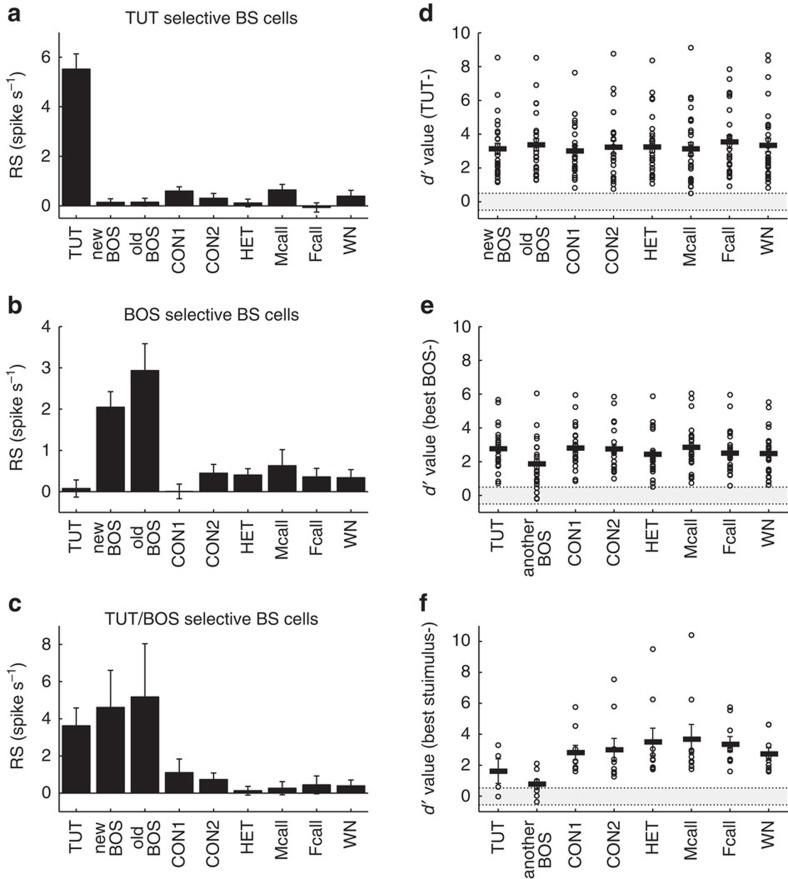
Sharply tuned auditory responses in song-selective BS neurons. (**a**–**c**) Mean RS (response strength) of TUT- (**a**), BOS- (**b**) and TUT/BOS-selective (**c**) BS neurons to each sound stimulus. The RS for tuned stimuli is significantly higher than those for all other stimuli. Mean±s.e.m., *n*=27, 26 and 9 for TUT-, BOS- and TUT/BOS-selective neurons, respectively, *P*<0.001 by Kruskal–Wallis test. (**d**–**f**) *d* values for best stimulus over other sound stimuli of TUT- (**d**) BOS- (**e**) and TUT/BOS- (**f**) selective neurons. The best stimulus for TUT neurons was the TUT. The best stimulus for BOS neurons was the old or new BOS, and the best stimulus for TUT/BOS neurons was either the TUT, old BOS or new BOS. Each open circle represents data from a single neuron. Black bars indicate mean *d* values. Grey areas indicate non-selective neurons (−0.5<*d* value<0.5). Note that all of the *d* value comparisons with unbiased stimuli are higher than 0.5.

In birds with tutor experience, subsets of BS neurons showed selective responses to experienced songs. Before tutor experience, no TUT-selective BS neurons were identified (0/29), whereas after tutoring in the same birds, 13% of BS neurons (27/207) were TUT selective (Fig. 2b). In isolated birds that lacked tutor song experience, TUT (paternal song)-selective BS neurons were rarely recorded (2/233) in the same age period, even though they had BS neurons that showed selective responses to other songs (17%, 39/233), suggesting that emergence of TUT-selective neurons depends on TUT experiences. The proportion of TUT-selective BS cells after tutoring was significantly higher than before tutoring, and also higher than in control birds (*χ*^2^-test, *P*<0.001, df=2). Notably, in addition to emergence of TUT-selective neurons, spike responses to TUT became precisely time-locked to specific syllables of TUT (Fig. 3c; Supplementary Fig. 4). TUT-selective neurons also showed nearly exclusive responses to TUT (Figs 3c,d, 4a,d, RS for the TUT and other songs, 5.50±0.64 and 0.29±0.09, respectively, mean±s.e.m., Kruskal–Wallis test, *P*<0.001, *n*=27 neurons, 10 birds). Among 27 TUT-selective BS neurons, 15 showed weak, but significant responses to one or two other sound stimuli (no. of other sound stimuli that evoked significant responses=1.9±0.4, mean±s.e.m.). The remaining 12 TUT-selective BS neurons responded exclusively to the TUT, which produced a very high SI (0.72±0.18, mean±s.e.m). Importantly, TUT-selective BS neurons did not respond even to the BOS, although in other brain areas important for song learning, called the ‘song system', responses to the TUT were usually accompanied by responses to the BOS^18^,^20^. In addition to TUT-selective BS neurons, a distinct subset of BS neurons showed exclusive responses to the BOS after tutoring. Previous studies have demonstrated that neurons in the song system show apparent BOS-selective auditory responses^18^,^21^,^22^,^23^,^24^,^25^,^26^. In the song system most neurons show responses biased towards the BOS; by contrast, we found that only 12% of BS neurons (25 out of 207 BS neurons) selectively responded to the BOS in the NCM after tutoring (Fig. 2b). Immature developing BOS, which were recorded at two different time points before electrophysiological recording started, were selected as song stimuli (old BOS and new BOS, recorded 47±8 days and 60±8 days (mean±s.d.)). Some BS neurons responded to either the old or the new BOS (10 out of 25 BOS-selective BS neurons), while other neurons responded equally well to both songs (5 out of 25). The remaining BOS-selective BS neurons (10 out of 25) showed selective responses to one type of BOS and weaker, but significant responses to other sound stimuli (no. of other sound stimuli that evoked significant responses=2.5±0.5, mean±s.e.m.). BOS-selective neurons were also recorded in isolated birds (11/233; new BOS selective: eight neurons, old BOS selective: two neurons, new/old BOS selective: one neuron) in the similar percentage with recordings in before tutoring birds (1/29). Those percentages were significantly lower than in after-tutoring birds (Fig. 2b; Supplementary Fig. 5, *χ*^2^-test, 0.01<*P*<0.02, df=2). As seen in TUT-selective neurons, BOS-selective neurons were highly biased towards the old and/or new BOS (Fig. 4b,e) regardless of tutor experience, thus yielding a high SI value in both tutored and isolated birds (0.67±0.18 and 0.75±0.16, respectively). Moreover, we found another subset of BS neurons (nine out of 207 BS neurons) that selectively respond to both the TUT and the BOS (TUT/BOS-selective neurons), but not to other stimuli (Figs 2b, 4c,f, RS for TUT, new BOS, old BOS and other stimuli; 3.62±0.96, 4.61±2.00, 5.18±2.86 and 0.51±0.14, respectively (mean±s.e.m.), Kruskal–Wallis test, *P*<0.001, *n*=9 neurons, seven birds), as reported in lateral magnocellular nucleus of the anterior neostriatum (LMAN) neurons^18^,^20^ in tutored birds. While the percentage of TUT/BOS-selective neurons in after tutored birds was small and there was no way to evaluate classification of selective neurons, it was significantly higher than that recorded in isolated control birds (1/233, *χ*^2^-test, 0.005<*P*<0.01, df=1). Using a microdrive, neurons were recorded from the NCM along with the dorso-ventral axis within a depth of 0.8–1.8 mm (Supplementary Fig. 1f). Song-selective neurons were found throughout the entire recording site with no correlation between the song type for which the neurons were selective, and their depth.

### TUT selectivity emerges with auditory not motor experiences

**Figure 5:**
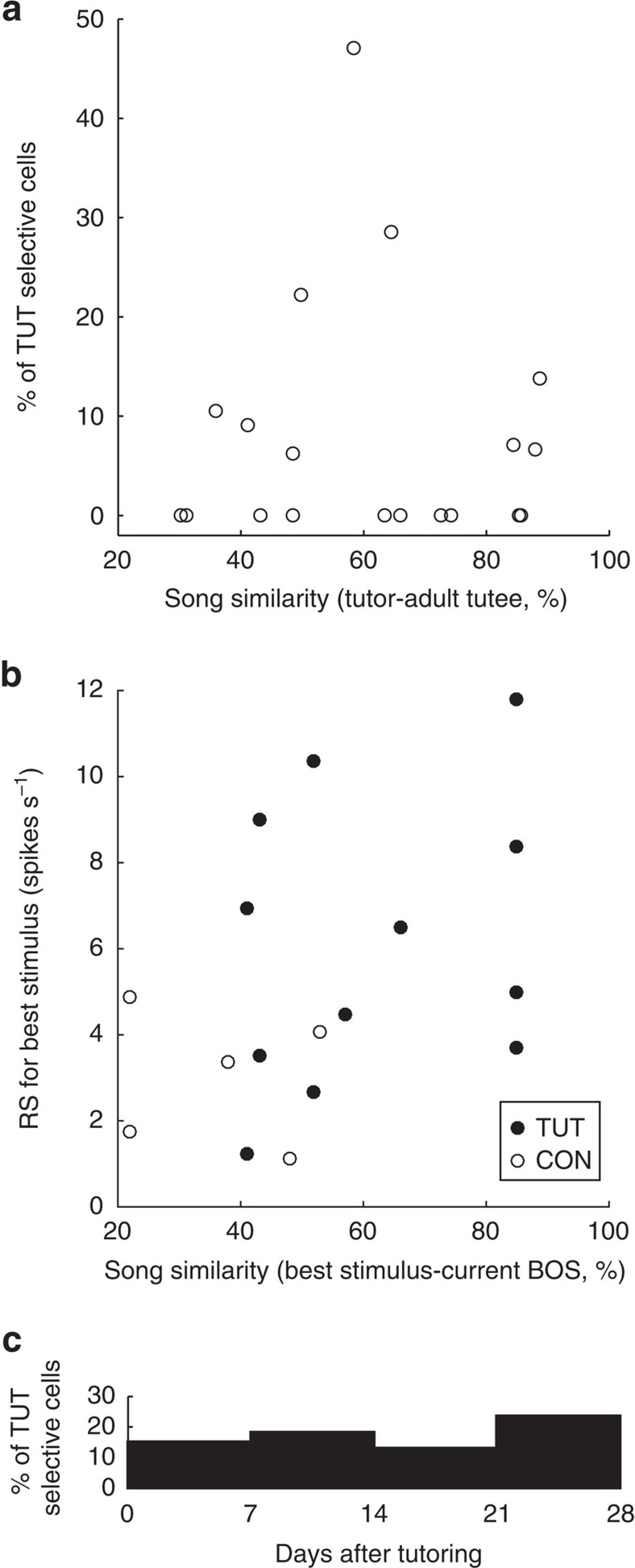
TUT-selective responses do not correlate with vocal learning from TUT. (**a**) The proportion of TUT-selective neurons recorded in a bird, is a function of song similarity between a bird's own song and the tutor song. There is no significant correlation between them (*r*=−0.02, *P*>0.1, *n*=19 birds). (**b**) Response strength (RS) to the TUT or CON in TUT-selective neurons (closed circle) or CON-selective neurons (open circle), respectively, in the function of song similarity between BOS, recorded ≤3 days before electrophysiology recording, and TUT or CON. There was no significant correlation between RS to TUT and similarity to TUT (*r*=0.27, *P*>0.1, 12 TUT-selective neurons from six birds), RS to CON and similarity to CON (*r*=0.15, *P*>0.1, 5 CON-selective neurons, four birds), and all RS to TUT/CON and similarity to TUT/CON. (*r*=0.42, *P*>0.05, 17 selective neurons, eight birds). (**c**) The proportion of TUT-selective neurons recorded among all BS-neurons recorded during each time period after tutoring (21 TUT-selective neurons, eight birds).

Neurons in the LMAN, which is necessary for song learning, respond to TUT in proportion to the amount of song learning, and not to transiently learned tutor songs^20^, suggesting that they respond to songs that are similar to their own vocalizations. We further assessed whether TUT-selective responses of BS neurons in the NCM reflect auditory experience, or the level of motor mimicking. The birds' songs became more similar to TUT along with the time after tutoring; however, the proportion of TUT-selective neurons found in each time period after tutoring was almost constant (Fig. 5c). Moreover, there was no correlation between the similarity of BOS (recorded within 3 days of electrophysiological recording) to TUT and RS to TUT (Fig. 5b), nor was there any correlation between the amount of song learning from TUT (similarity at the final song) and the proportion of TUT-selective neurons in a given bird (Fig. 5a). Furthermore, there was no correlation between the similarity of BOS to the stimulus song (CON) and the RS to that song (Fig. 5b). These observations suggest that auditory selective responses to a specific song in NCM do not represent vocal motor learning. Rather, it suggests that TUT-selective responses represent auditory TUT experiences and the memories formed from those.

**Figure 6:**
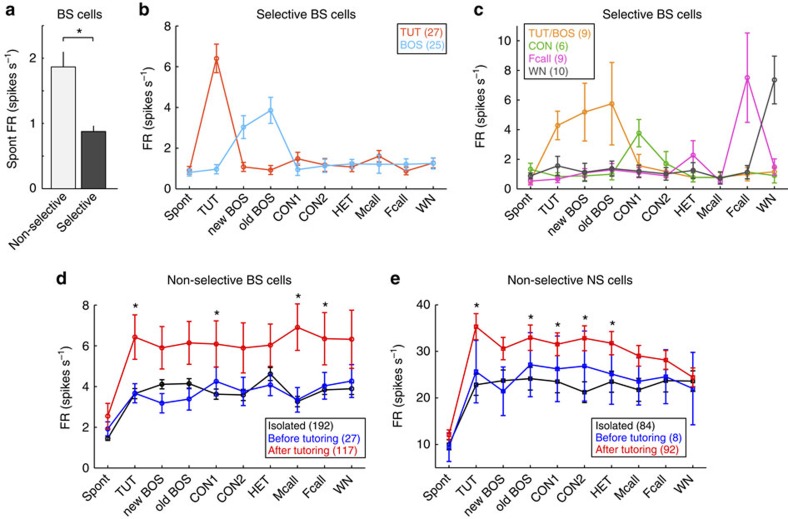
TUT experience alters neuronal activity of song-selective BS neurons. (**a**) Spontaneous firing rates (FR, spikes s^−1^) of non-selective and selective BS neurons. (Mean±s.e.m., *n*=337 and 132 for non-selective and selective BS neurons, respectively, *P<0.001, Mann–Whitney *U*-test). (**b**,**c**) Mean spontaneous firing rates (Spont) and firing rates during presentation of each song stimulation, in song-selective BS neurons (TUT and BOS-selective BS-neurons (**b**) and TUT/BOS, CON, Fcall and WN selective neurons (**c**), mean±s.e.m.). Numbers in parentheses indicate numbers of neurons. (**d**,**e**) Mean spontaneous firing rates (Spont) and firing rates during presentation of each song stimulation, in nonselective BS neurons (**d**) and NS neurons (**e**), recorded before tutoring (blue), after tutoring (red), and in isolated (black) birds. Asterisks indicate significant differences between isolated and after-tutoring birds (*P*<0.05, Tukey–Kramer's multiple comparison test).

We then investigated how auditory experience altered physiological properties of NCM neurons. After TUT experience, about a half (43%, Fig. 2b) of BS neurons showed selective neuronal responses to specific song stimuli. Interestingly, the spontaneous firing rate of these selective BS neurons was significantly lower than that of non-selective BS neurons, regardless of the song stimulus to which each neuron selectively responded (Fig. 6a–c). Selective neurons have lower spontaneous firing rates and responded exclusively to a specific song, whereas non-selective neurons responded equally well to all stimuli with higher spontaneous firing rates. Furthermore, with tutor experience, both non-selective BS-neurons and NS neurons showed greater auditory responses to the song stimuli (Fig. 6d,e). Interestingly neither decreased spontaneous firing of selective neurons nor increased auditory responses in non-selective neurons were provoked by auditory experience with birds' own songs (isolated birds). This implies that auditory experience with TUT (not their own songs nor other vocalizations) alters and differentiates a subset of selective neurons to form a memory for song learning.

### GABA circuits sharpen song selectivity in the NCM

**Figure 7:**
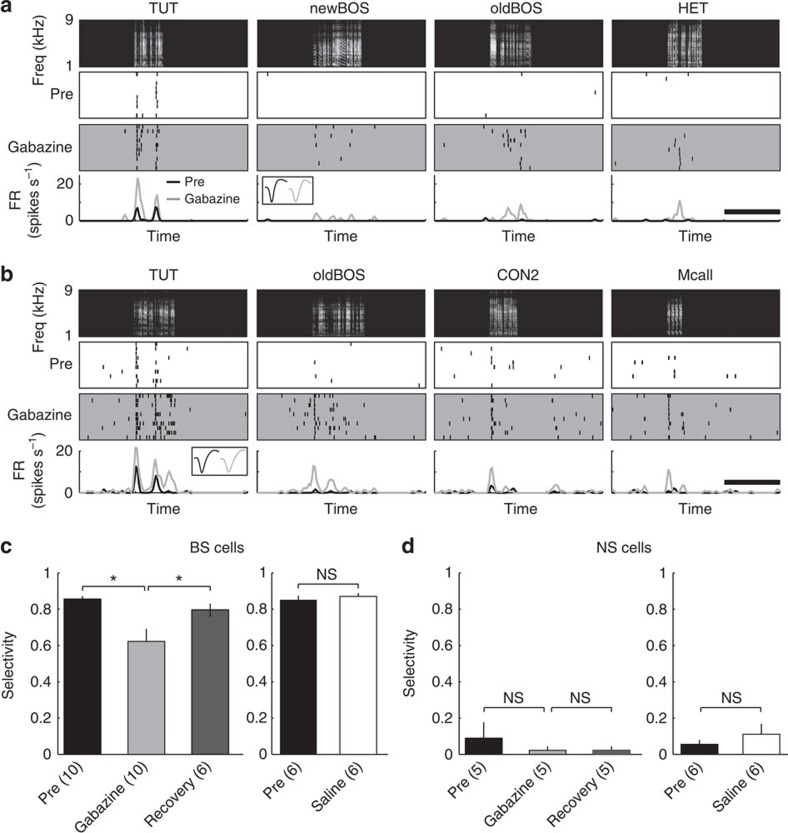
Blocking GABAergic inhibition decreases selectivity in BS neurons. (**a**,**b**) Representative auditory responses of TUT-selective BS neurons before and after gabazine injection. Neural responses to each sound stimulus before and after gabazine application are shown with raster plots (middle) and mean firing rate (FR) curves (bottom) and are time-aligned with spectrograms of sound stimuli (top). Inset: mean spike waveforms before (pre: black) and after gabazine (grey) injection. Scale bars, 2 s (**c**,**d**) Mean selectivity index before and after gabazine (left) or saline control (right) injection in BS (**c**) and NS (**d**) neurons. Data for Pre, gabazine/saline and recovery are summarized 10 min before and 20 and 60 min after gabazine/saline injection. BS neurons exhibited significantly lower selectivity after gabazine injection (left panel), whereas saline injection did not affect selectivity (right panel). By contrast, gabazine had no effect on NS neurons, which show less selectivity. Numbers in parentheses denote the number of neurons in each group. Mean±s.e.m. *P<0.005 by Wilcoxon signed-rank test.

Selective neuronal responses have been well characterized in sensory cortical networks^27^,^28^, and increases in response bias from less biased inputs by local GABAergic inhibitory interneurons have also been reported^29^. In the zebra finch auditory cortex, the NCM, approximately half of the neurons are GABAergic^30^, and blocking GABAergic inhibition results in alterations in song-evoked responses^17^,^31^. Moreover, decreased spontaneous firing rates in selective BS-neurons with TUT experience might be caused by recruiting tonic GABA inhibitory inputs. Thus, we examined the role of GABAergic inhibition in experience-dependent selective responses to learned songs in the NCM by blocking GABAergic inhibition with local injections of the GABA_A_ receptor antagonist, gabazine. We found that after local gabazine injection (200 nl of a 1 μM solution in saline), TUT-selective BS neurons became responsive to other song stimuli that originally did not evoke significant responses, though the responses still remained biased to TUT (Fig. 7a,b). Similarly, BS neurons selective for the BOS, TUT/BOS or other sounds decreased their response selectivity after local GABA inhibition was blocked, whereas a control saline injection did not affect response selectivity (Fig. 7c, left panel, SI, pre, 0.86±0.02, gabazine, 0.62±0.07, recovery, 0.80±0.03, Wilcoxon signed-rank test, *P*<0.005, *n*=10 neurons, 6 birds, right panel, pre, 0.85±0.02, saline, 0.87±0.02, *P*>0.05, *n*=6). Neuronal responsiveness increased after gabazine injection with both preferred and non-preferred stimuli (RS for preferred stimulus, pre, 3.76±1.10, gabazine, 4.62±1.12, 0.3>*P*>0.1, RS for non-preferred stimulus, pre, 1.26±0.46, gabazine, 2.14±0.42, *P*<0.05, Wilcoxon signed-rank test (one-tailed)). Gabazine application did not change the spontaneous firing rate in these neurons (pre, 0.51±0.24, post, 0.61±0.26, *P*>0.2). This suggests involvement of non-selective inhibitory auditory input into selective neurons that masks small non-preferred auditory responses, as seen in the sensory tuning of cortical neurons^29^,^32^,^33^. In contrast, gabazine injection did not change response selectivity in non-selective NS neurons (Fig. 7d, left panel, SI, pre, 0.09±0.09, gabazine, 0.02±0.02, recovery, 0.02±0.02, Wilcoxon signed-rank test, *P*>0.05, *n*=5).

### Arousal state modulates song selectivity

**Figure 8:**
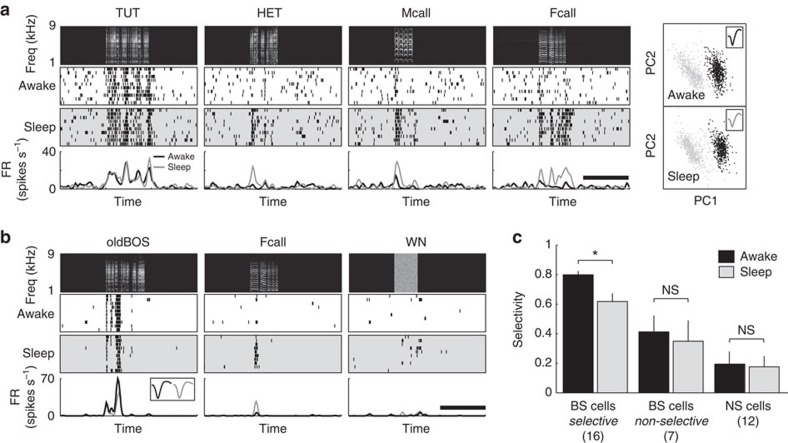
State-dependent modulation of song selectivity in the NCM. (**a**,**b**) Representative auditory responses of TUT- (**a**) and BOS-selective (**b**) BS neurons during wakefulness and sleep. Neuronal responses during wakefulness and sleep are shown with raster plots (middle) and mean firing rate curves (bottom) time-aligned spectrograms of the sound stimuli (top). Spike clusters (right panels in **a**) and spike waveforms (inset) during wakefulness and sleep states did not differ. Scale bars, 2 s (**c**). The selectivity index during wakefulness and sleep states in song-selective and non-selective BS neurons, and NS neurons. Song-selective BS neurons exhibited significantly lower selectivity during sleep than wakefulness, whereas selectivity did not change between wakefulness and sleep in non-selective BS neurons and NS neurons. Numbers in parentheses denote the number of neurons in each group. Mean±s.e.m., *P<0.01 by Wilcoxon signed-rank test.

In mammalian sensory cortices, sensory processing is markedly influenced by an animal's arousal state^34^,^35^. Furthermore, a recent study showed that anaesthesia leads to an imbalance of excitation and inhibition in the primary visual cortex, making orientation tuning under anaesthesia broader than during wakefulness^36^. By contrast, in songbirds, neurons in brain areas that are necessary for song production and learning respond to BOS playback under anaesthesia or during sleep, but do not respond in awake animals^26^,^37^,^38^,^39^,^40^,^41^,^42^. We investigated whether and how song selectivity in the NCM is modulated by arousal state. We found that arousal states strikingly altered the selectivity of NCM neurons in a cell-type-specific manner (Fig. 8). Song-selective BS neurons responded to a greater number of song stimuli while birds were sleeping normally under darkness (Fig. 8a,b). This state-dependent selectivity modulation was seen in selective BS neurons regardless of the song to which the neurons were tuned, but it was not observed in non-selective BS or NS neurons (Fig. 8c, SI, selective BS neurons, awake, 0.80±0.02, sleep, 0.62±0.05, Wilcoxon signed-rank test, *P*<0.01, *n*=16 neurons, 9 birds, non-selective BS neurons, awake, 0.41±0.11, sleep, 0.35±0.14, *P*>0.05, *n*=7, NS neurons, awake, 0.19±0.08, sleep, 0.18±0.07, *P*>0.05, *n*=12). Thus, selectivity was strikingly modulated by behavioural states in a cell-type-specific manner.

## Discussion

Here, we provide electrophysiological evidence that a memory of an experienced tutor song is represented in a subset of auditory cortical neurons in zebra finch brain. To the best of our knowledge, this is the first report demonstrating sharply tuned tutor song-selective neurons in the juvenile songbird brain during natural behaviour. Following auditory experience with a tutor song, we found that a subset of BS neurons in the NCM exhibited lower spontaneous firing rate and highly selective auditory responses to the tutor song, suggesting that auditory experiences shape auditory cortical circuits of juvenile birds and that the memory of the tutor song is represented in a small, specific subset of neurons.

Our findings strongly support the notion that the NCM is a storage site of tutor song memory. Various studies using different experimental approaches have suggested that the NCM is a potential locus of tutor song memory. Blockade of molecular signalling pathways in the NCM prevent normal vocal learning in juvenile birds^8^, and lesions in the NCM in adult birds disrupt behaviours related to tutor song memory^9^,^10^. Electrophysiological recordings from awake, head-restrained birds show a different ratio of habituation in multi-unit responses to repeated presentations of the tutor song and conspecific songs^14^,^15^,^16^. Furthermore, there are positive correlations between the strength of song learning and the level of immediate early gene expression in the NCM upon hearing a tutor song^12^,^13^. Here, we demonstrate that after tutor song experience, a small subset of neurons of one cell type in the NCM showed highly selective auditory responses to the experienced tutor song. We also found another subset of neurons of the same cell type that selectively responded to the BOS, in strong contrast to the song system, where most neurons show a biased response to the BOS. Neither the proportion of TUT-selective neurons recorded in NCM, and RS to TUT showed correlation with the amount of vocal learning from TUT. This suggests that emergence of TUT-selective neurons is caused by TUT auditory experience, and not by vocal motor learning. Furthermore, TUT, but not BOS auditory experience decreased the spontaneous firing of one subset of BS neurons. On the other hand emergence of selective neurons itself did not depend on TUT auditory experience as BOS-selective and other song-selective neurons were also found in isolated control birds in the higher percentage than in before tutoring birds. These results suggest that in addition to normal development, TUT song, not BOS auditory experience, modifies intrinsic neuronal properties of selective BS-neurons or local GABA inhibitory circuits to form a TUT memory. These neurons modified by auditory experience might regulate later motor learning. Further experiments will be necessary to identify possible mechanisms for differentiating selective neurons in which auditory memories are held, and to determine how TUT memory within them is associated with subsequent motor learning.

**Figure 9:**
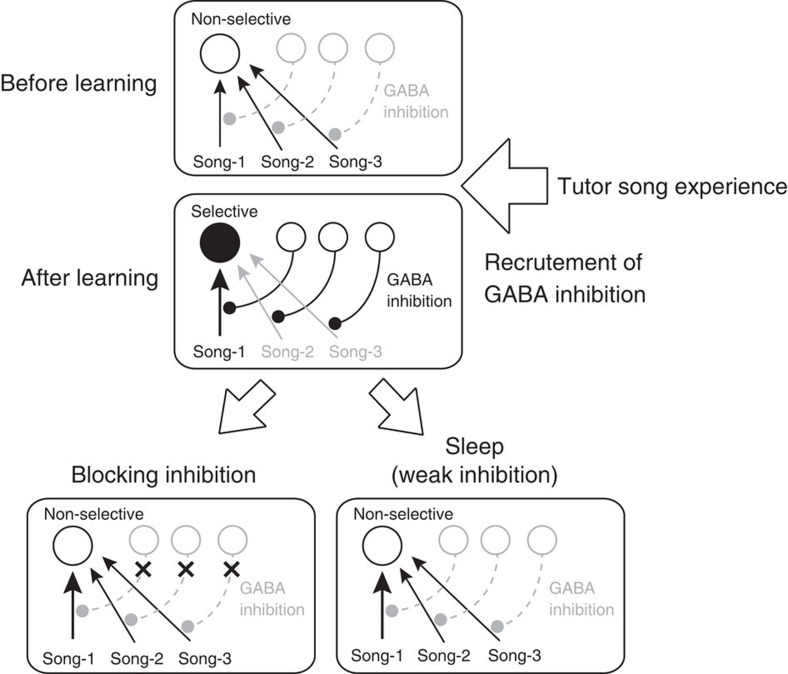
Experience-dependent GABA inhibitory circuit recruitment in zebra finch auditory cortical circuits. Before learning, a BS neuron (open circle) receives various non-selective auditory inputs (song-1, song-2 and song-3). At this stage, GABA inhibition is still immature. Tutor song experience matures GABAergic inhibitory circuits, which suppress unbiased auditory inputs (song-2 and song-3), leading to selective responses to a song experienced by the bird (song-1) in a specific BS neuron (closed circle, after tutor song experience). After blocking GABAergic inhibition or during sleep, which would cause weak inhibition, the song-selective BS neuron becomes responsive to other auditory inputs.

In mammalian sensory cortices, GABAergic inhibition plays an important role in sensory tuning^29^,^32^,^33^, and the balance of excitation and inhibition regulates the temporal precision of spike responses^43^. Moreover, GABAergic transmission, as well as GABA-mediated inhibitory short-term plasticity in the auditory cortex, matures in relation to auditory experience^44^,^45^. During early development, experience-dependent refinement of the excitation–inhibition balance occurs in the primary auditory cortex and contributes to maturation of cortical circuits^46^. As in primary auditory cortical neurons in mammals, we show that selective auditory response in higher order auditory cortical neurons, acquired as a result of TUT auditory experience, is regulated by local GABA inhibition in zebra finches. Pharmacological blockade of local GABA inhibition decreased the response selectivity of song-selective BS neurons without changing their spontaneous firing rates, though their responses still remained biased. That suggests involvement of non-selective inhibitory auditory input to TUT-selective neurons (Fig. 9), while slightly biased responses after GABA blockade still suggests possible involvement of biased afferent inputs (either by weakening of non-preferred or by strengthening of preferred input). Together, here we suggest that common features of sensory experience-dependent neuronal plasticity in cortical circuits^1^,^2^,^3^ occur with TUT auditory experience in the zebra finch auditory cortex, and that GABA-mediated sensory tuning contributes to auditory template formation in zebra finch song learning. Further studies on TUT auditory experience-dependent GABA circuit development in zebra finch auditory cortex would provide us further insights.

In this study, we also showed that song-selective BS neurons were markedly modulated by the arousal state of the bird. Song-selective BS neurons showed decreased response selectivity while the birds were asleep. Previous studies have shown that behavioural states strongly modulate auditory responses in song system nuclei. Neurons in the HVC and RA in the song system, as well as those in the NIf, interface of the auditory and vocal motor systems^47^, robustly respond to the BOS during sleep or under anaesthesia, but not during wakefulness^26^,^37^,^38^,^39^,^40^,^41^,^42^. Gating of auditory responses by arousal state can be seen in the selective response to the BOS within song system nuclei, but not in the primary auditory cortex (field L), where neurons do not show selective responses to a specific song^37^. Here, we found state-dependent modulation of selectivity of auditory responses in the NCM. Song-selective BS neurons showed markedly decreased selectivity while birds were asleep. On the other hand, responsiveness to each song itself, not selectivity, increases during sleep, consistent with previous studies. In mammalian sensory cortices, behavioural states markedly modulate sensory processing^34^ and regulate activity of inhibitory interneurons in a subtype-specific manner, which consequently leads to modulation of animal behaviour^48^. Furthermore, a recent study of the mouse primary visual cortex showed that anaesthesia (or sleep) leads to an imbalance between excitation and inhibition, making orientation tuning during the anaesthetized state broader than that during wakefulness^36^. Similar mechanisms of brain state regulation of cortical activity might explain increased responsiveness in the NCM. Direct or indirect inputs from the NCM to the song system can also modulate responses there. Norepinephrine regulates brain state of arousal, attention and alertness^49^,^50^. Norepinephrine infusion modulates auditory response measured by immediate early gene expression in the NCM^51^. Further study is needed to deeply understand mechanisms by which neuromodulators regulate brain state-dependent auditory responsiveness in the NCM^35^.

Our study provides clear electrophysiological evidence of the presence of sharply tuned tutor song-selective neurons, representing auditory memory of TUT in the higher auditory cortical area of juvenile zebra finches. We also suggest how early auditory tutor experience shapes cortical circuits of avian brain during song learning. At this stage, it remains unclear whether tutor song-selective or BOS-selective neurons in the NCM contribute to later sensorimotor learning. To determine how these song-selective neurons interact with other interrelated brain areas to guide sensorimotor learning will be an important future direction toward understanding neural mechanisms underlying sensorimotor learning in early development.

## Methods

### Subjects and experimental design

Experiments were performed in accordance with experimental protocols approved by the Animal Care Committee at Okinawa Institute of Science and Technology (OIST) Graduate University. Twenty-five male juvenile zebra finches (20 tutored and 5 isolated), born and reared in our colony, were used in these experiments. Birds were raised in cages with their parents and siblings. Then, at 10–12 days of age, the father was removed, and siblings were reared with their mothers in sound-attenuating boxes until 31–35 days of age, when they were individually isolated. Thereafter, male juvenile birds were housed individually in sound-attenuating boxes, and at 53–62 days of age, birds underwent surgery for electrode implantation. After recovering from surgery, single-unit activity was recorded to determine auditory responsiveness (before tutoring). After several neural recordings, juvenile birds were housed together with a tutor bird for 2 weeks, except during the period of electrophysiological recordings (tutored birds). So as not to disrupt learning of the tutor song, neural recordings were started more than 5 days after tutoring, and were continued until birds reach adulthood (80±8.6 days) (after tutoring). Five birds were kept isolated, and periodically recorded electrophysiologically (islolated birds). We also performed electrophysiological recordings in 6 juvenile birds (54–85 days of age) that were reared with their parents (father and mother) and siblings in our colony room until 50 days of age, and then isolated in recording boxes. Electrophysiological data obtained from these birds, with respect to the ratio of tutor song-selective or BOS-selective neurons, were comparable to those from birds that were isolated and then tutored. Thus, data from colony reared birds were included in the same analysis.

### Surgery and electrophysiological recording

We recorded single-unit neuronal activity from the NCM in freely behaving male juvenile zebra finches^52^. Two tetrode wire electrodes (nichrome wire, diameter, 12.5 μm,impedance, 300–500 kΩ, Kanthal Palm Coast) and one reference electrode attached to a microdrive were implanted into the NCM using stereotaxic coordinates (anterior: 0.5 mm, lateral: 0.5 mm from the bifurcation of the sagittal sinus) under isoflurane anaesthesia (1.3%), and fixed to skulls with dental cement. After recovery from anaesthesia, juvenile birds can freely move with implanted electrodes. Juvenile birds were wired to the amplifier only during neuronal activity recordings. Neuronal recordings were done 5–8 h per day, every day from 4 days before until 38 days after tutoring, except the first 1–5 days of tutoring, to give juvenile birds enough time to learn the song. Using a microdrive, recordings were obtained from 0.8 to 1.8 mm in depth. To assess bird arousal states, we monitored EEG activity from the brain surface through a stainless steel wire. Power spectral density of EEG signals was calculated using Welch's method, and sleep state was defined by occurrence of strong EEG power at low-frequency range (less than 10 Hz)^53^. Most of the time, single-unit neuronal activity was recorded in awake behaving birds, and in some cases, neuronal units were also recorded in the same birds while they were asleep (35 units from 11 birds, aged 55–87 days). Neuronal signals were amplified 5,000 or 10,000-fold and EEG signals were amplified 1,000-fold. Neuronal and EEG signals were band-pass filtered at 0.5–9 kHz and 0.7–170 Hz, respectively, digitized (40 and 20 kHz for neuronal and EEG signals, respectively) with the Plexon MAP system, and stored on a PC. Sound stimulation was presented from a speaker, and neuronal responses were recorded simultaneously with the presentation of sound stimuli. We also monitored each bird's vocalizations during electrophysiological recordings. Spike sorting was performed off-line using the Offline Sorter (Plexon), and well-isolated single units were submitted to subsequent analysis with MATLAB (MathWorks). After completion of electrophysiological recordings, electrical lesions were made (20 μA for 20 s), and recording and drug injection sites were histologically verified (Supplementary Fig. 1e,f).

### Song recording and analysis

BOS were recorded using an Avisoft recorder (Avisoft Bioacoustics). The BOS was recorded twice interval of >6 days (old BOS, 49±9 days, new BOS, 64±9 days, mean±s.d.). These recordings were used as song stimuli for electrophysiological experiments. Song stimuli included a TUT, the old BOS, a new BOS, 2 different conspecific adult zebra finch songs (CON1, CON2), a HET, an adult male zebra finch call (Mcall), an adult female zebra finch call (Fcall), and white noise (WN). These were presented 10 times from a loudspeaker in a semi-random order with an inter-stimulus interval of 4 s. To evaluate the degree of vocal learning after tutoring, song similarity of a bird's song recorded at adulthood (135±5 days of age), and their tutors' songs were measured (%similarity) using SAP 2011 (ref. 19). For each bird, 10 song motifs were measured for their similarity to the tutor's song motif, which was used for song playback during electrophysiological recordings, and averaged (Supplementary Fig. 1c). For isolated birds, song similarity to their genetic father's song motif, which was also used for electrophysiological recordings, was measured.

### Electrophysiological data analysis

Neurons that showed a significantly higher firing rate during the song stimulus than during the pre-stimulus baseline period (*P*<0.05, Wilcoxon signed-rank test (two-tailed, unless otherwise noted)) during at least one song stimulation were submitted to further analysis. We classified neurons based on the mean spike width and the duration from negative to positive peak, using 30 spike examples for each neuron. The auditory responses of each neuron were quantified by response strength (RS), which represents the difference in mean firing rate during the sound stimulus and during a pre-stimulus baseline period. To further assess the response bias between two sound stimuli, we calculated *d* values using the following equation:


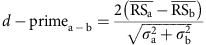


where 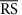 is the mean response strength to the stimulus and *σ*^2^ is the variance of the RS (ref. 18). As in previous studies^54^,^55^, *d*-prime>0.5 was used as a criterion for biased response, since it represents an RS to the biased stimulus >2 × as great as that to other stimuli^18^. If *d* value comparisons between a specific song and all other songs were greater than 0.5, the neuron was categorized as selective for that specific song. In cases where the absolute *d* value for TUT-BOS was <0.5, and a neuron exhibited significant responses to both TUT and BOS (new BOS and/or old BOS), but not to others, a neuron was classified as ‘TUT/BOS-selective' (Fig. 4c,f). We further assessed to how many song stimuli each neuron responded to by calculating the selectivity index: 1−(*n*/total number of song stimuli), where *n* is the number of auditory stimuli that drive statistically significant auditory responses (*P*<0.05, Wilcoxon signed-rank test).

### Pharmacological experiments

To assess the role of GABAergic inhibition in neural responses, the GABA_A_ receptor antagonist gabazine (SR95531, Sigma-Aldrich) was locally injected during neural recordings. For drug injection experiments, a cannula was implanted along with the tetrodes. Gabazine was used at 1 μM because that concentration did not induce seizure-like neural activity in our preliminary experiments. The single-unit activity was isolated, and auditory responses to the song stimuli were recorded. Then, a bird was placed in the stereotaxic apparatus, and gabazine (200 nl of a 1 μM solution in saline) was locally injected through the guide cannula over a period of 5 min (KDS 310 Nano Pump, KD Scientific). The bird was returned to the recording chamber, and auditory responses of the same single unit were again recorded at 20 min (gabazine condition) and at 1 h after injection (recovery condition). In control experiments, the same amount of saline was injected, and electrophysiological recordings were performed before and after injection.

### Data availability

All relevant data are available from the authors on request and/or are included with the manuscript.

## Additional information

**How to cite this article:** Yanagihara, S. & Yazaki-Sugiyama, Y. Auditory experience-dependent cortical circuit shaping for memory formation in bird song learning. *Nat. Commun.* 7:11946 doi: 10.1038/ncomms11946 (2016).

## Supplementary Material

Supplementary Information Supplementary Figures 1-5
