# Supplementary material for: Auditory experience-dependent cortical circuit shaping for memory formation in bird song learning
Source: Nat Commun. 2016 Jun 21;7:11946. doi: 10.1038/ncomms11946 (PMC4919517; doi:10.1038/ncomms11946)
Supplement: Supplementary Information — Supplementary Figures 1-5 [file ncomms11946-s1.pdf]

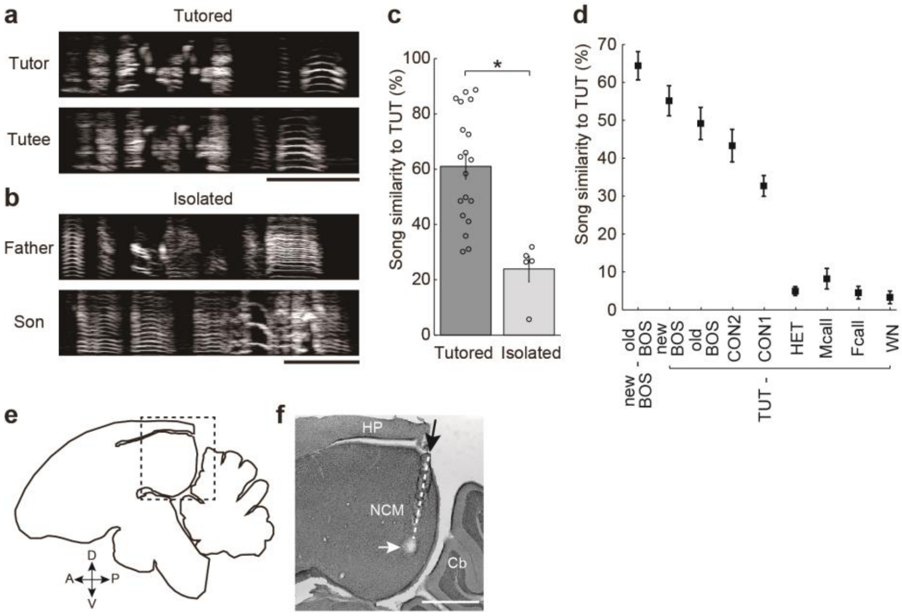

Supplementary Figure 1 Verification of experimental procedures.

a, Sound spectrogram of tutor (upper) and its tutee's song at adulthood (129 DPH) (lower). Their song similarity was 72.6%. Scale bar: 200 ms **b**, Sound spectrogram of isolated birds song at adulthood (125 DPH) (lower) and his father's song (upper). Their song similarity was 26.8%. Scale bar: 200 ms **c**, Song similarity to the tutors' song (TUT) in tutored and isolated birds (n = 19 and 5, respectively). Similarity to TUT in tutored birds is higher than that in isolated birds (Mean ± s.e.m., *P<0.005, Mann-Whitney U test). **d**, Song similarity between TUT and presented song stimuli (new BOS vs old BOS, TUT vs other songs) used for neural recordings (Mean ± s.e.m.). **e, f,** Histological verification of recording sites. Schematic drawing of sagittal section of a zebra finch's brain (**e**). f, A photograph Nisslstained brain section, around the area shown with dotted square in **e**, for identifying recording site in NCM (**f**). The white

arrow shows a site of electrolytic lesion made with the recording electrodes to verify the electrode position in NCM. White dotted line indicates the electrode track penetrated from dorsal to ventral. The black arrow indicates direction of the penetration. NCM: Caudomedial nidopallium, HP: Hippocampus, Cb: Cerebellum. Scale bar: 1 mm

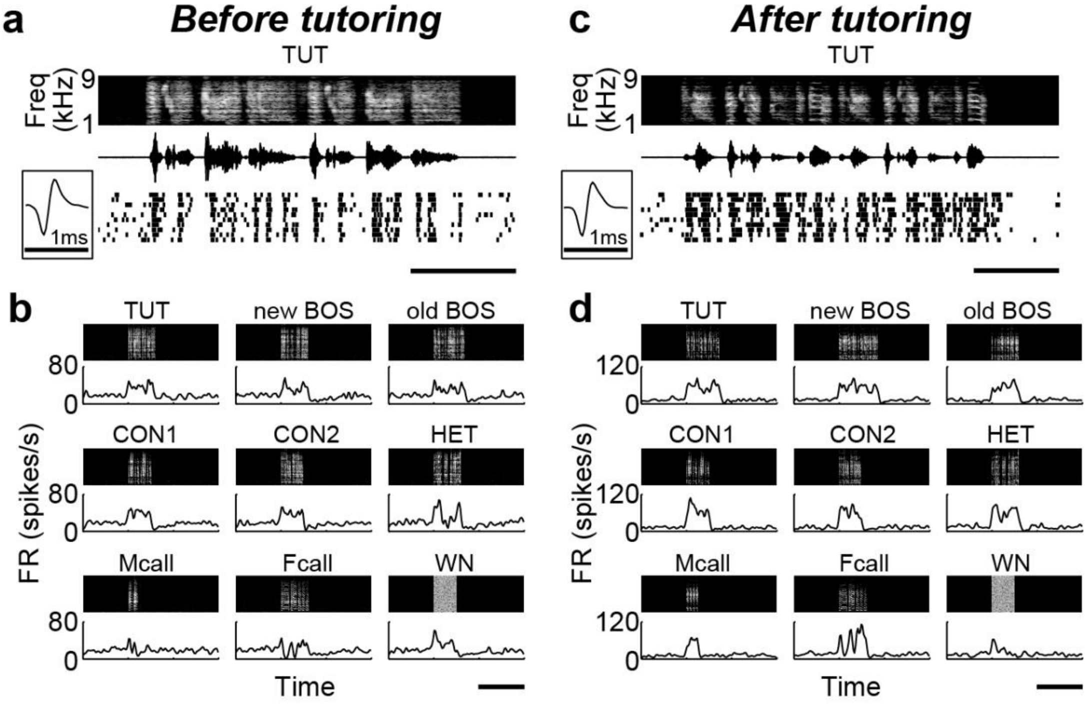

Supplemental Figure 2 NS neurons remain non-selective even after tutoring.

A representative non-selective NS neuron recorded before (55 days of age) (**a, b**) and 22 days after tutoring (79 days of age) (**c, d**). The neural responses to playback of the tutor song (TUT) are shown as a raster plot (bottom) time-aligned with the TUT spectrogram (top) and oscillogram (middle) (**a, c**). Scale bars: 400 ms, Inset: mean spike waveform. Mean firing rate (FR) curves for each auditory stimulus (**b, d**). Note that NS neurons responded most of the stimulus equally well non-selectively both before and after tutoring. TUT: tutor song, BOS: the bird's own song, CON: conspecific song, HET: heterospecific song, Mcall: male call, Fcall: female call, WN: white noise. Scale bars, 2 s

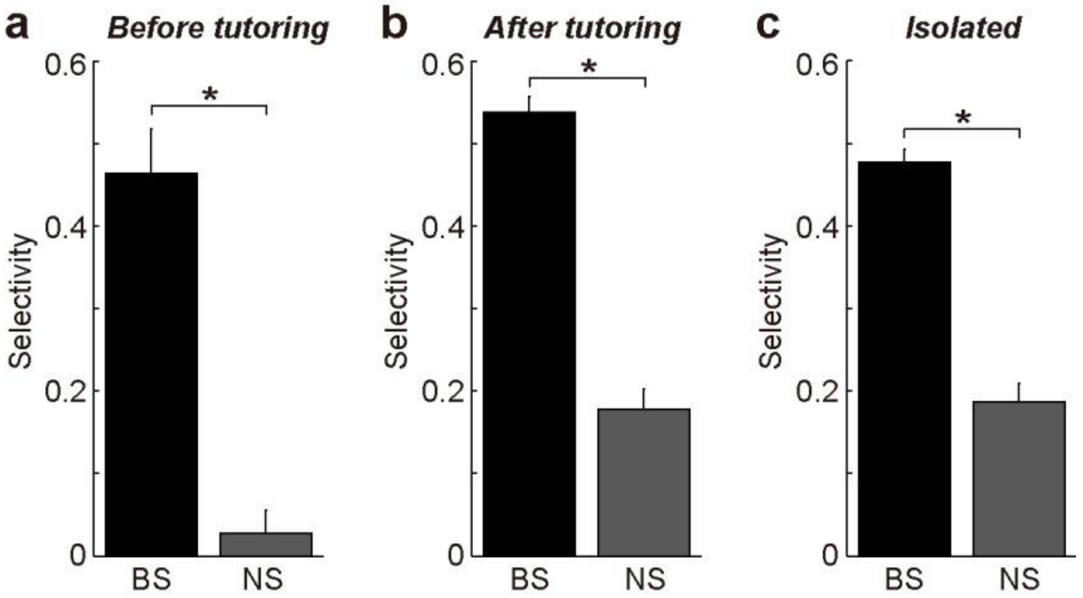

Supplementary Figure 3 Song selectivity is higher in BS neuron than NS neuron regardless of TUT song experience. Song selective index (1 - (n/total number of song stimuli)) is greater in BS means more selective, than in NS neuron in all conditions. Before tutor song experience: BS neuron 0.46 ± 0.05, NS neuron 0.03 ± 0.03, after tutor song experience: BS neuron 0.54 ± 0.02, NS neuron 0.18 ± 0.02, isolated: BS neuron 0.48 ± 0.02, NS neuron 0.19 ± 0.02, mean $\pm$ s.e.m., $p < 0.001$, Mann-Whitney U test).

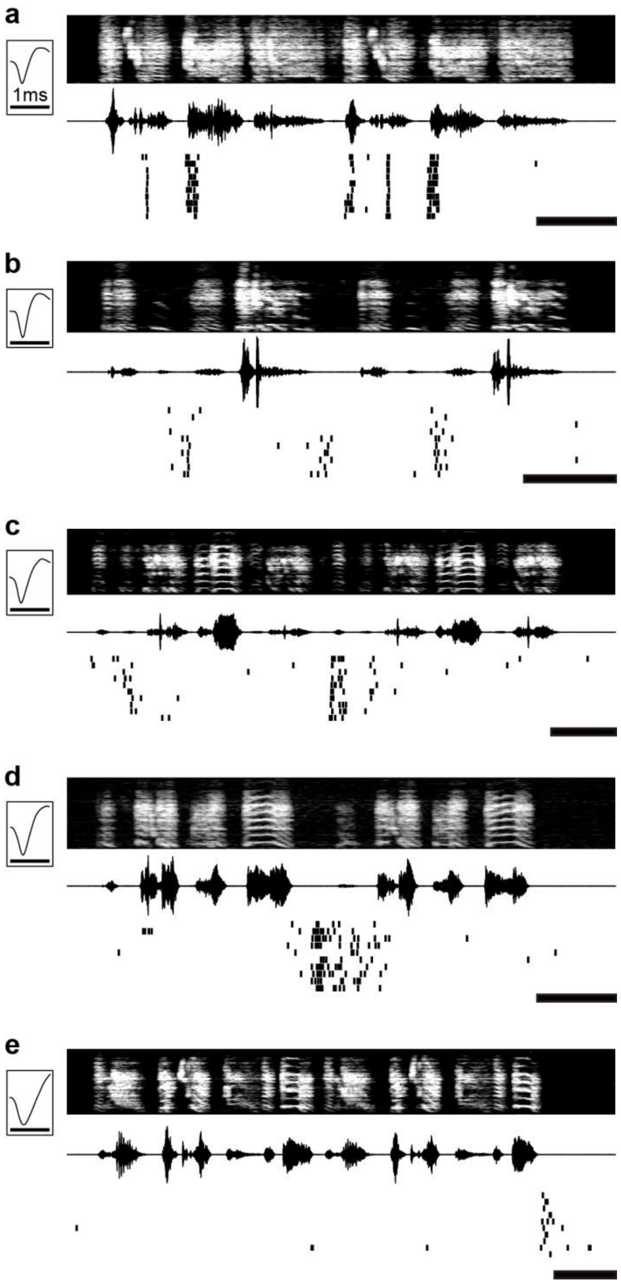

Supplementary Figure 4 Time-locked spike response in TUT-selective BS neurons.

Representative TUT-selective BS neurons recorded from 5 tutored birds at 9 days (64 DPH) (**a**), 14 days (64 DPH) (**b**), 24 days (71 DPH) (**c**), 27 days (78 DPH, colony reared) (**d**) or 27 days (84 DPH) (**e**) after tutoring. Inset: mean spike waveform for each neuron. Scale bars: 200 ms

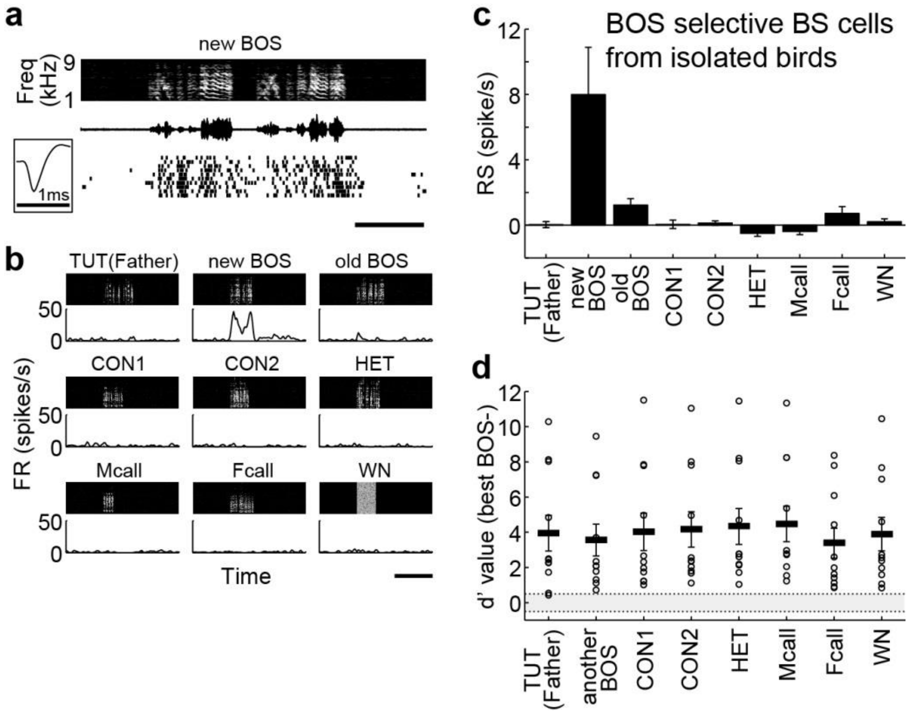

Supplementary Figure 5 Sharply tuned auditory responses in song-selective BS neurons in isolated birds

a, A representative BOS-selective BS neuron recorded from an isolated bird (69 days of age). Neural responses to playback of the new BOS are shown as a raster plot (bottom) time-aligned with the new BOS spectrogram (top) and oscillogram (middle). Scale bar: 400 ms **b,** Mean firing rate (FR) curves for each auditory stimulus. TUT: tutor song, BOS: the bird's own song, CON: conspecific song, HET: heterospecific song, Mcall: male call, Fcall: female call, WN: white noise. Scale bar: 2 s **c,** Mean RS (response strength) of BOS-selective BS neurons to each sound stimulus. The RS for tuned stimuli is significantly higher than those for all other stimuli. Mean ± s.e.m., n = 11 BS neurons from 4 isolated birds, $P<0.001$. **d,** D-prime values for best stimulus (old or new BOS)

over the other sound stimuli of BOS-selective BS neurons. Each open circle represents data from a single neuron. Black bars indicate mean d-prime values. Gray areas indicate non-selective (-0.5 < d-prime value < 0.5).
